# After Coal: Meanings of the Durham Miners' Gala

**DOI:** 10.3389/fsoc.2020.00032

**Published:** 2020-05-15

**Authors:** John Tomaney

**Affiliations:** Bartlett School of Planning, University College London, London, United Kingdom

**Keywords:** coal mining, place, class, Durham, miners

## Abstract

The annual Durham Miners' Gala (or “Big Meeting”), first held in 1871, was a distinctive feature of the coalfield throughout its productive life. It became an important date in the calendar of national labor movement. But it was also a festival of music and banners. The Durham Miners' Association represented a crucial component of the British working class and a cornerstone of Laborism. But its distinctive class practices also marked out a region (see Cooke, 1985) with particular political traditions. The last mine closed in 1994. Yet the Gala has survived the demise of industry and the conflicts and struggles which it produced. In July 2019, the 135th Gala attracted tens of thousands of people. This paper reviews the existing academic literature on the Gala and draws on a range of sources (including memoirs, novels, paintings, films, and biographies) to chart the shifting cultural history of the Gala. I show that the form and content of the Gala has changed throughout its history and has been freighted with a range of meanings. The Gala can be seen as an example of “intangible cultural heritage” in which knowledge of the past aspects of working class and communal life are reproduced and reimagined even when the material basis for them has apparently disappeared.

## Prolog

The Durham Miners' Gala presents a contemporary paradox. Established by the Durham Miners' Association (DMA) in the nineteenth century, during the twentieth century it became one of the largest annual trade union demonstrations in Europe, attracting hundreds of thousands of people. By 1994, in the aftermath of the bitter 1984/5 miners' strike, all the coalmines in County Durham had closed. Declining attendances had been a feature of the Gala since the 1970s as employment contracted but, by the first decades of the twenty-first century, it was resurgent, attracting tens of thousands of participants. Originating as a political demonstration, at the heart of the Gala is the parading of banners and bands. Traditionally, the members of each DMA Lodge (union branch) and their families walked alongside their banner and band, through the streets of the city of Durham, to a great gathering by the River Wear; a format that, in its essence, remains unchanged. This performance inspires the Gala's popular appellation, “The Big Meeting.” In 2019 the Gala attracted its biggest crowd for decades. The mines have gone but rituals associated with mining life linger.

The Gala today appears to some as an anachronism. The journalist James Bloodworth, who visited in 2016, saw it as “a carnival of nostalgia,” functioning “as something like a historical re-enactment society,” highlighting how “when the past becomes an obsession it can act as a dead weight on meaningful action in the present” (Bloodworth, 2016). Celebrating a dead industry and its way of life is backward-looking. Nostalgia signals a failure to adapt. But from the beginning, the bands and banners, and the parade itself, represented not just the occupational power of the miners but also a particular form of localism: “they marched through with friends and family as a village” (Beynon and Austrin, 1989, p. 80). Jack Lawson (1881–1965), Durham miner and later Labor MP and minister in the Attlee government, claimed that for the people of the Durham coalfield, the Gala was less a political demonstration than “the spontaneous expression of their communal life” (Lawson, 1949, p. 161). It is this aspect that has gained greater prominence in recent years and helped to transform the Gala.

Bloodworth's arguments bring to mind the long history of efforts to erase the past in the name of progress and modernity. In a debate in the House of Commons in 1879 that concerned early efforts to preserve the megaliths at Stonehenge, the Tory MP Francis Hervey dismissed them as “absurd relics of our barbarian predecessors” (quoted in Bender, 1993, p. 113), although this disavowal failed to stem the tide of conservation nor, much later, the emergence of an entire heritage industry. As early as 1974, the then director of the Northern Arts Association, responding to claims that cultural policy should reflect regional identity, declaimed:

I'm not from these parts. I'm from the Home Counties. I regard my mission as bringing the arts to the North. Northumberland is dead, and so is its so-called folk culture. So are the pits' (quoted in Quartermain, 1990, p. 10).

The death knell of the Gala has been sounded periodically over recent decades but, so far, it has refused to die. It proves impossible to draw a neat line under the past but the question here is, what is the meaning of the Durham Miners' Gala after the end of coal mining?

In this paper I consider why the Gala persists despite the demise of the coalfield. The paper proceeds inductively. I explore the anthropology, historical sociology, cultural history, and political philosophy of the Gala. I use secondary data in the form of novels, song, poetry, paintings, film, journalism, memoir and biography. I draw from the cultural record to show how the Gala has been a forum for shifting and complex identities which are explored heuristically in relation to themes of work, community, and place. While the Gala began as a place for the representation of industrial and political power of the miners, it always expressed much more than this. I show how, more recently, the communal aspects of the Gala have come to the fore as the industry has vanished, while its performance gives expression to a *genius loci*. Among the several ironies that emerge is how nostalgia for an earlier, better, nobler Gala is observable almost from its birth. In light of this account, I set out a framework for interpreting the development of the Gala, investigating arguments about nostalgia, collective memory, and intangible culture before concluding with an assessment of the contemporary meanings of the Gala.

To dismiss the Durham Miners' Gala as mere nostalgia is to misunderstand the occasion and misinterpret nostalgia itself. The Gala expresses a collective memory of the coalfield and the ways of life its supported. It represents intangible cultural heritage. It is living history. It expresses an identity located in place. These are not static. The historical evolution of the Gala reveals that its purpose and role have been perennially contested but essential aspects, notably the parading of bands and banners, as an expression of identity, forms its essential core. The Gala began as a manifestation of the growing industrial power of the Durham miners, but from the beginning it was also a gathering of the diverse communities that formed around the pits. Its enactment in Durham symbolized a reordering of regional life and still signals the complex relationship between the city and surrounding towns and villages that mirrors broader social divisions between cities and towns. It was both a demonstration and celebration that gave expression to the particular political traditions of the coalfield, notably the moderate Laborism embodied in the practices of the DMA. An extraordinary range of artifacts record, emplace, and reflexively interpret the Gala and shed light on its history and shape its meanings. These are examined below in relation to work, community, and place.

## Industry, Community, and Place

### Industry

The original purpose of the Miners' Gala was to express the nascent power of the Durham miners. That power grew and became politicized during the twentieth century and the Gala's form and content shifted accordingly. The Durham miners fought epic struggles in the nineteenth century to form a union and advance their interests in the face of brutal repression by the coal-owners (or “masters”). The formation of the DMA, at the Market Tavern in Durham in 1869, represented a great victory. At same time, though, the coalfield was characterized by paternalism on the part of owners and conciliation on the part of the miners (Beynon and Austrin, 1994). It occurred alongside the “canceling of the bond,” which overturned the system of annual servitude under which the miners had labored since the eighteenth century. The first act of the new union was to commit to the holding of a “Yearly Demonstration” (Wilson, 1907, p. 31) which symbolically replaced the annual hiring and expressed the rising industrial power of the union.

The first Gala was held on 12th August 1871. The prospect of thousands of miners from surrounding villages arriving in the city filled the local bourgeoisie with trepidation. Although surrounded by the largest and most productive coalfield the world had ever seen, Durham was an Anglican ecclesiastical and university citadel, its skyline graced by a magnificent Norman Cathedral and medieval castle. The DMA was led by Primitive Methodists whose religious traditions were a rebuke to pretensions of the Church of England. The first Gala was held tentatively in Wharton Park, then on the edge of the city. Thereafter, subsequent Galas were held on the Racecourse within sight of the Cathedral, signaling the miners arrival at the center of the region's cultural and political life. Although it drew on a “well-established pattern of mass assembly” (Moyes, 1974, p. 26) stretching back to the great gatherings that greeted John Wesley across the county in the eighteenth century and which accompanied efforts to establish a union earlier in the nineteenth century, the first Gala inaugurated a new era.

The Gala originated in the assertion of the “rights” of the Durham miners to adequate pay, fair terms and conditions. It was a demonstration of the potential power of the miners, but it was far from a riotous assembly. On the contrary, the DMA leaders wanted their members to display sobriety, responsibility and restraint in their behavior and demands. And indeed, they did. Reporting on the second Gala, which took place on 15 June 1872, the *Durham Advertiser* praised the discipline and respectability of the event:

As they left the field there was one striking circumstance, in connection with which was apparent to everyone, as these men – some of them far advanced in life, others in the prime of manhood, and young boys who could not have been long from school – passed through crowded thoroughfares, and that was their orderly conduct throughout. A more well behaved body as they appeared on Saturday could no well be found. There was an entire absence of all drunkenness and no ill-spoken word never offended the ear of the spectators in the streets. This indeed speaks volumes for the class of men who occupy such an important position in connection with our northern trade and commerce. It is evident that the pitmen are looking to higher aims and objectives than the groveling practices and pursuits of previous generations – practices which still linger in some of our pit villages and mining districts among the less educated. As the miners entered the city so they took their departure. Their conduct producing a most favorable impression and demonstrating beyond dispute that even in such a large number of men, whose lives are devoted to incessant toil, they may be benefited and improved by a closer union with their fellows to attain the common objects by fair and legitimate means. The pitman has become a respectable and peace loving citizen and his conduct proved so on Saturday (quoted in Temple, 2011, p. 17).

Early Lodge banners attested the moderation of the miner's cause, their willingness to compromise with the coal-owners for better pay and conditions. Primitive Methodists were disproportionately represented in the leadership of the Lodges and the early officials of the DMA. Many banners contained religious allusions and Biblical references (Moyes, 1974). Sacriston Lodge banner enjoined, “Do ye unto all men as ye would they should do unto ye,” after Matthew 7: 12, *KJV*. The Monkwearmouth banner bade, “Come let us reason together,” after Isaiah 1: 18, *KJV*. The leaders of the DMA appealed to reason and the better Christian nature of the coal-owners. Strikes were to be avoided. The tone of local politics was set for a century. Reporting on the 1872 Gala, the *Durham Chronicle* described how,

The display of banners was a very prominent and pleasing feature of the demonstration. Altogether there are were upwards of 70 flags on the ground. They were ranged round the full length of the field near the water's edge and thence across the field opposite to that on which the platform had been erected and it is hardly necessary to state, composed as they were of every imaginable color and hue, that they formed as they fluttered in the breeze a very pretty and imposing spectacle. The greater proportion of them were indeed artistic productions both in design and execution whilst a few owed a great deal of their beauty and attractiveness to elaborate trimmings with which they had been adorned. Nearly all of the flags had been specifically provided for the occasion and in nearly every instance the representations as well as their spirit of the various mottoes, sentiments, couplets, etc., were creditable alike to the heads and hearts of the colliers evincing as they did a fair, conciliatory and even kindly feeling toward their employers (quoted in Moyes, 1974, p. 34).

In the late nineteenth and early twentieth centuries the Gala was a place to air the great political and industrial issues of the day, including the relationship with the Labor Party, the advancement of trade union rights and the case for the nationalization of the coal industry (Temple, 2011; Mates, 2016). Before the 1890s speakers included radicals such as Joseph Cowen (1873), Peter Kropotkin (1882), Annie Besant (1884), and Tom Mann (1887). The atheist MP Charles Bradlaugh spoke 10 times at the Gala. In 1888, the Fenian John O'Connor Power was speaker. In 1887 Tom Mann used his speech to attack the opposition of the DMA leaders to parliamentary legislation to create an 8-h day, returning in 1899, 1900, and 1901 to repeat his. Later speakers included James Larkin (1914) and the Communist MP Shapurji Saklatvala (1928). While the presence of radical speakers enlivened the Gala, they did not shift the traditions of moderation of the DMA as a whole.

Tensions between the rank and file and union leadership—personified during this period by John Wilson, simultaneously General Secretary of the DMA and Liberal MP—were played out at the Gala. Lodges nominated speakers for the Gala who were chosen by ballot. In particular, it became a battleground between the aging union leadership which had advocated conciliation and was attached to the Liberal Party, and the rising Independent Labor Party, which attracted younger members and wanted a more assertive union that advanced its sectional interest. This drawn out process lasted until the First World War when the DMA shifted its allegiance from the Liberals to the nascent Labor Party. The shift was reflected in the changing content of banners from a Liberal-Methodist influence into a discernibly Labor-Socialist form and more secular images became the norm (Beynon and Austrin, 1989; Wray, 2009a,b). In his novel, *The Big Meeting* (1967), David Bean evokes the scene at New Elvet, stressing the centrality of the parading of banners,

Banner followed banner. Some were militant, with nineteenth century allegorical pictures of miners and their families before and after union, of noble-looking miners besting tyrant top-hatted coal-owners looking like Sir Jaspers. There were others with portraits of miners' champions – Nye Bevan, Keir Hardie, Arthur Cook, local lodge heroes too, even one with Marx and Lenin and the slogan Workers of all Lands Unite!

One or two [banners] were draped with black crepe … that meant during the past year a man, or possibly a number of men, had been killed down that lodge's pit (Bean, 1967, p. 232, 233).

Over time, political tensions and contradictions were resolved on the banners. Speaking at the Gala in 1952, Nye Bevan told how he had observed a banner earlier in the day which bore images of Peter Lee (1864–1935) and Arthur Cook (1883–1931), two Miners' leaders who “used to quarrel like Kilkenny cats” (quoted in Temple, 2011, p. 125). The banners contributed to an understanding that, over time, contested positions could be incorporated in a broadly-based labor movement.

The defeat of the General Strike and the year-long Miner's Lockout in 1926 led to the marginalization of radical politics in the Durham coalfield. Erstwhile left-wing Lodge leaders such as Will Lawther (1889–1976) and Sam Watson (1898–1967) abandoned their previous militancy to join the ranks of the moderate officials, reinforcing the DMA's reputation as bastion of the right-wing in the labor movement (Garside, 1971). After serving in other official positions, Watson became General Secretary of the DMA in 1946, swiftly centralizing power and seeking, among other things, to exercise greater control over the Gala speakers. Watson's rise to power coincided with the nationalization of the industry in 1947 and the Gala, according to the miner-historian, David Temple, was transformed into a “state occasion” (Temple, 2011, p. 116). The high demand for coal to fuel reconstruction meant the DMA held extraordinary political clout in the new post-war political settlement and Watson saw the Gala as a means to both display and exercise the power of a free trade union. In Watson's words, the Gala was “an anvil upon which the purpose and meaning of the union could be hammered out” (quoted in Beynon and Austrin, 2014, p. 479). Intimate links with the Labor Party meant that leading politicians were prominent among the speakers, but Watson also extended his patronage beyond the Labor movement. The US Ambassador, Averill Harriman, was invited as guest to the 1946 Gala and successive Israeli and Yugoslav ambassadors became regular attenders (Temple, 2011). According to Watson's widow, Jenny,

The Gala was the highlight of the year. It was the highlight of my husband's life. It was a fantastic political platform. It was faithfully recorded in all the newspapers and the radio. There was only a small platform before Sam became General Secretary; but it grew and it was deliberate. The men in the coalfield, at the coalface, picked the speakers, of course, but Sam was forever asking around for the guests. As I say, it was a major political platform, and Sam realized that (quoted in Beynon and Austrin, 2014, p. 478–479).

For 20 or 30 years, the Gala became critically important in the calendar for leaders of the Labor Party. Hugh Dalton, MP for Bishop Auckland and Chancellor of the Exchequer in the Attlee Government, was a regular attender (Pimlott, 1985). Recalling the 1947 Gala, Dalton wrote, “Never in all the years had there been such a rally of bands, banners and sturdy and confident humanity in this old city” (Dalton, 1962, p. 143). For Dalton (1962, p. 238) the Gala was “always a deeply moving performance.” Michael Foot, who was later to become leader of the Labor Party but then a left-wing firebrand recalled,

I started there in 1947. That's when I shared the platform with Arthur Horner. It was strange because the Durham area emerged as a right wing within the union and the Labor Party, but I was elected to go there regularly. The Durham Miners' Gala is a fine occasion today, taking place as it does in that beautiful city. But in those days it was absolutely sensational. There were so many lodges you see and they had to start bringing them in at half past eight in the morning. The whole city absolutely throbbed with the thing from early in the morning right through until you left. And you left absolutely drunk with it – the music, the banners and all in that beautiful city. It overwhelmed you really. In those days it was, far and away, the best working-class festival that there was in this country. Far and away the best. It was just marvelous (quoted in Beynon and Austrin, 2014, p. 478–479).

By the time Sam Watson retired from his position in the DMA in 1963, however, the coalfield was already facing decline. Shifts in energy policy in the 1960s led to large-scale colliery closures and associated job losses. The Gala during this period became a place to mourn the death of pits as banners were paraded ostensibly for the last time. David Bean's novel, *The Big Meeting* (1967) concerns the final Gala for the “Sharlstone” miners whose colliery is slated for closure. The Durham coalfield played a key role in the English folk revival during this period, notably at the Birtley Folk Club (Mitchell, 2014). Harraton colliery—known local by its nickname “Cotia” pit—produced two of the great songsmiths of the era in Jack Elliott and Jock Purdon. Their most famous songs lamented pit closures and the perceived betrayals that accompanied them (see Lloyd, 1978). In “Farewell to ‘Cotia,” Jock Purdon sang bitterly of the pit's impending demise,

Ye brave bold men of ‘Cotia,

The time is drawing thus,

You'll have to change your banner, lads,

And join the exodus.

In “The ‘Cotia Banner”, Purdon sings,

Unfurl the ‘Cotia banner, boys! Where are the rebels bold

That marched behind the banner on Gala days of old?

Ed Pickford's song, “A strange lover is a coalmine”, is infused with nostalgia for lost work, camaraderie and bygone Gala days,

Joe survived ‘til he grew old

But he never did forget about the coalmine

He loved the coal and the Union too

He had a miner's pride about his own time

I remember the hot Julys

The banners that flew in the Durham skies

I remember the hopes and the fears and the lies

What a strange lover is a coalmine.

(http://www.ed-pickford.co.uk/strangeloverisacoalmine.html).

In his song, “And I Shall Cry Again,” Alex Glasgow evokes the feelings of a generation which would not go down the mine but nevertheless felt moved by the spectacle of the Gala,

And I shall cry again, and not know why again.

The silken banners summon up the tears,

The men who march beneath them touch the soul.

I have not known the pitmen's hopes and fears,

I learnt them from the books I read at school.

But I shall cry again, and not know why again.

(https://www.youtube.com/watch?v=qs3759NSP14).

By 1973 only 27 banners were paraded at the Gala and its future began to be questioned. The DMA, however, doggedly stuck to the idea that the Gala was principally an occasion for miners. In 1979 the Durham County Association of Trade Councils wrote to the DMA asking if other unions could be included in the Gala. The DMA official replied,

I need not remind you that the Big Meeting is uniquely a Miners' Gala … As you will gather, we are jealous of our traditions and our Big Meeting … If you understand our traditions you will have half expected a ‘no' (quoted in Barron, 2010, p. 267).

The Gala entered a limbo until the 1984/5 miners' strike which signaled the beginning of the end for the coal industry, but also created the conditions for its reinvention.

### Community

According to Cohen (1985, p. 118), “People construct community symbolically, making it a resource and repository of meaning, and a referent of their identity.” There are two spatial scales at which community operates in relation to the Gala. At one scale, the Gala gives expression to the County as a focus for identity, but villages were the base communities of the Durham coalfield. Collieries also existed in large urban settlements such as Sunderland and South Shields but, at the time of nationalization in 1947, production was decentralized in 234 collieries (https://www.nmrs.org.uk/mines-map/coal-mining-in-the-british-isles/durham-coal/) mainly located in small villages hastily constructed in the second half of the nineteenth century. Warnings against the romanticizing community are commonplace in the academic literature (e.g., Bourke, 1994; Amin, 2004) and Gilbert (1995, p. 51) argues that mining communities should be seen as “constructed and contested places.” Conflicts in the mining industry were always embedded in communal contexts (Smith, 1980; Barron, 2010). In the Durham case, particular forms of communal life were constructed to which the Gala gave expression.

Although the Gala was held each year by the DMA, the union's authority lay in the hands of its Lodges which, in some cases, had preceded the formation of the union itself. Lodges operated as “a pit level parliament,” presided over by the elected Lodge secretary (Brown, 1987, p. 139, Moyes, 1969; Douglass, 1977). Decisions were taken collectively by ballot of members. In relation to the workplace a key activity was the organization of quarterly ballots (“cavils”) that determined the allocation of workplaces underground, and hence miners' earnings potential, that were a unique feature of the Durham coalfield. Although the mono-industrial structure might be seen as giving rise to natural solidarities, the Lodge was a place in which sharp differences were reconciled. Crucial cleavages formed that lasted well into the twentieth century notably around religion—between Anglican, Methodist (Primitive, Wesleyan, etc.) and Catholic—and attitudes to temperance: “While other institutions, such as chapel, church, Co-operative Society and the workingmen's club, provided rival foci to the union, the lodge provided a point of unity amidst a complex network of influences” (Brown, 1987, p. 147; Tomaney, 2018).

Election to Lodge office was to assume a leadership role in the wider community. The Lodge involved itself in the activities of the village such as the allocation of housing, the supply of coal or the provision of water, managed the distribution of welfare benefits in relation to unemployment, sickness or compensation for industrial injuries, and the levies which supported the Durham Aged Mineworkers' Homes Association. As the franchise was extended and the role of local government grew at the district and county level, representatives of the Lodges began to play a leading role in electoral politics. In many villages Lodge officials also controlled the local Cooperative Societies. Peter Lee, the last of the great Primitive Methodist leaders, held the position of checkweighman at Wheatley Hill colliery, membership of the Parish Council, Easington Rural District Council and, later, chairmanship of Durham County Council, DMA Agent and President of the Miners' Federation of Great Britain, leading Lawson (1949, p. 130) to describe him: “chief of the civic life of Durham and leader of its people.”

In 1934 in *An English Journey*, Priestley (1934) painted a memorably grim portrait of village life in Shotton but his account overlooked the forms of self-organization that shaped these communities. In his account of the Durham village, “East Tanthope,” the sociologist Benney (1946, p. 56) noted that, “wherever, by chance, the eye rests upon some building more attractive than its neighbors, one almost invariably finds that it owes its existence to the organized efforts of the miners themselves. Their clubs, welfare institutes, and co-op stores are outstanding institutional buildings.” For the miner turned novelist, Sid Chaplin (1916–1986), the Gala was the place where the villages came together to honor a sense of social achievement. Reflecting on his first visit to the Gala in the 1930s, he wrote,

In those days there were about 150 pits, of course, 150 bands, 150 banners, and it was a great and glorious sight. Each one represented the village, and each village was, in fact, a sort of self-constructed, do-it-yourself counter-environment, you might dub it. The people had built it themselves” (Chaplin, 1978, p. 80–81).

Chaplin acknowledged the imperfections of the society in which he grew up, such as the gendered nature of opportunities they offered, and admitted that his greatest ambition as a youth had been to escape, but he insisted, “their achievements cry out for celebration” (Chaplin, 1978, p. 81, 71). For Benney (1946, p. 122), as far as “East Tanthope” was concerned,

Nothing has come easily to this village. When it felt a need, it had tried to supply it for itself, and if anyone opposed the effort, the village had fought. Every institution in the village with the exception of the cinema, the post-office and the church, the people had built themselves or struggled for through their union. Their community, forged deep underground in dark stony places, drew on elemental sources of strength and discipline. Personal ambition was tamed to the Lodge office, the committee table, the pulpit and the craft of the pit.

The banner was a crucial mode of identification because, it “provided the unifying element through which the village life became established as ‘community.' It had to embrace all the village; this was its purpose” (Beynon and Austrin, 1994, p. 224). The Gala was “patterned in the village life and custom” (1989, p. 71). According to Chaplin (1978, p. 81),

So the banner, which was so beautiful, represented something very solid and very substantial indeed. And while one recognizes that all things have to go, and mining as it was in the North East has gone, and in many ways gone for good, one also has to recognize that there was a great achievement and the greatest of these achievements was that with the poorest of materials, in the poorest of circumstances, fighting a battle underground and fighting a battle against bad housing and bad sanitation on the surface and poor wages, people banded together and built in their villages little communities which were quite something to live for.

In his memoir, *A Man's Life* (1932), Jack Lawson offers an exhilarating account of a city bedecked in banners carried like regimental flags. For Lawson, while the Gala was created to advance the political interests of the miners, by the 1930s, it had been transformed into a festival of a proud culture in which families played a central role. In *Peter Lee*, his biography of the interwar miners' leader, Lawson suggested that great political speeches were now less important than the celebratory aspects of the Gala,

It is to gather round these platforms the vast mass is supposed to have come. The meetings play a great part, but to tens of thousands they are merely incidental, for it must be confessed the Big Meeting means much more than that. It is now more an institution than a meeting: more social than economic. It is a combination of all the Miners' Lodges, but the vast family eclipses everything (Lawson, 1949, p. 163).

Lawson (1932, p. 240) described the Gala as “a kind of social baptism.” The pageant introduced each generation to its history and identity. Years later, the Durham miner, Dave Douglass, recalling the Galas of his youth in the 1950s and 1960s, saw the occasion as “a living museum” that provided lessons in the history of struggle and reform:

Year after year at the Durham Gala since boyhood you watched the epic stages of our history parade by on magnificent banners. Each scene was explained as the banner came up, and then again as it did rounds of the big field, then again as the banner marched away – and this every year from the shoulders of your dad through to adolescence, year after year this kind of thing was reinforced (Douglass, 1981, p. 62).

The Gala as carnival was a theme from the earliest times. A lyrical and nostalgia-infused account of the Gala held on July 28th, 1923 appeared in the *Durham Advertiser*.

“The hail-fellow-well-met atmosphere introduced by these bright-eyed lights, these comfortable dames, these men of mature ways, yes, and even the really old men, was a delight never to be forgotten. As they pushed and danced their way through the narrow streets they glowed with the very spirit of carnival, living for one short day in the world of forgetfulness. The cares of home were left behind and only the joys of the moment reigned supreme. The good many hands lent an air of gaiety and yet perhaps an air of sadness to the proceedings, for who can hear good music and not be compelled to look back down the vista of years and feel just a tinge of longing? And the banners, to the passers-by just a pretty, painted piece of silk, but meaning so much to those who follow them, standing for unity and comradeship, and all that unity and comradeship means. Some of these colors were draped as a gentle tribute to some fellow man who would never again partake of the joys of the Gala; and who could look upon the old men laughing and dancing without realizing the singular appropriateness of such institutions as the aged miners' homes, just a something which will help to keep the light of laughter in those eyes? Just a something which will help to bring some measure of comfort to those old heroes. Yes it was a carnival – just a carnival no thought or word of strike, of blood, or revolution, just a carnival” (quoted in Temple, 2011, p. 80).

In such accounts, the Gala resembles more the Mardi Gras in New Orleans, or the fiestas which honor the relics of Catholic saints which occur in the Mediterranean or Latin America.

The communal aspects of the Gala grew in importance after the 1990s and resulted in the very obvious growth in the visibility of women at the Gala as organizers and participants, as musicians and bearers of the banners. The gendered nature of participation in the Gala was taken for granted by many of those involved for much of its history. Dave Douglass recalls:

…once a lodge reached a meeting place, the mams picked a corner of the field which was theirs, and picnics were begun, and throughout the day it was a rallying point for the dads coming back from the pubs and the committee men coming back from the speeches (Douglass, 1981, p. 62).

It is the proliferation of banner groups across the county which underpins the resurgence of the Gala. Notably, women take a prominent role in these Groups. In 2019 there were 60 banner groups in Durham. While they incorporate a range of views regarding their role in the community, they share a common aim of maintaining the Gala and “keeping the show of nostalgia on the road” (Ross Forbes, personal communication, December 2019). The effort that goes into this is considerable, not least in raising money to refurbish and maintain historic banners and to create new ones. Banners themselves have always tended to be commissioned from professional makers. There has also been a revival of the brass band culture, previously associated with the Lodges. There are 27 bands across the county that parade regularly at Gala. Strikingly, while the earliest photos of the Gala show the bands as entirely male, today they often led and dominated by women and have a marked intergenerational character. In the aftermath of the death of the coal industry the community aspect of the Gala has grown to become its critical component.

The Esh Winning Colliery Banner Group was formed in 2005. The group sought to retrieve the original banner from Beamish Museum. It worked with professional conservators to restore the banner, seeking Heritage Lottery Funding, with the aim of displaying it in the local social club. At the same time the group commissioned a replica banner to parade at the Gala. In 2006, local clergy blessed the new banner the evening before it was paraded. In the account of Rendell et al. (2010, p. 126): “the banner was the heart of their community and a physical representation of the pride in Esh Winning as a place.” In the case of the New Herrington Banner Group, a diverse membership, including people with no direct links to the coal industry, saw their activity not as act of remembrance or heritage, but as a way of maintaining community identity in a period of uprooting. The Group, its banner and its participation in the Gala contributes to “emotional regeneration”; memories of the past provide succor and inspiration for a community seeking a post-industrial future (Stephenson and Wray, 2005, p. 87). Bennett (2016) identified similar ambitions in the village Gala held in Wheatley Hill in 2007, albeit in ways that marginalized some stories and experiences in pursuit of a collective identity.

Scott (2009, p. 67) claims that, historically, the banners were concerned with “the representation and symbolization of the working man as hero, and if this was not delivered through male figures it was expressed in a catalog of other symbolic images designed to appeal to this sentiment.” But this presents a partial image of the iconography of the Gala. As Wray (2009a) shows, lodge banners reflect a range of representations of occupation, community and trade union that make overlapping, and even contradictory, claims about politics, industry and welfare and memorialize the struggles for improvements in each field. The “red banners” of Chopwell, Follonsby, and Bewick Main present images of Marx and Lenin, but these were unrepresentative of the majority, which typically called for reform and nationalization of the industry rather than the overthrow of capitalism. Welfare demands figured prominently. The Rainton Adventure Lodge banner carried an image of the Durham Aged Miners' Homes, the housing association established by workers' subscription. Some banners carried images of the colliery itself but typically in its wider setting, signaling “the symbiotic relationship between community, mine and trade union” (Wray, 2009a, p. 160). Nevertheless, the new generation of banners gives greater prominence to images of community (Scott, 2009). The DMA itself is seeking to transform its purpose from an industrial to community union in which the importance of place looms large (Wray, 2009a), albeit a community identity “based on a shared industrial past” (Mellor and Stephenson, 2005, p. 343).

### Place

An important dimension of the performance of the Gala concerns its siting in the city of Durham. Although generally overlooked in academic treatments, the theme of place emerges strongly from the range of artistic production inspired by the Gala. In his memoir, *A Man's Life*, Jack Lawson locates the spectacle in the topography of the landscape and the morphology of the city

Above the fluttering banners, the old square Castle, on its foundation of rock, rises clear cut against the sky. seeming to block further passage that way. But the procession moves on. and as it passes slowly over the bridge one can see the tree shadows like etched pictures in the seemingly still waters of the river below. Gradually the marchers wedge themselves into the narrow street which is called Silver, and past the might squat Cathedral, 'Halfchurch of God, half' castle 'gainst the Scot', standing there so gray and quiet in its own grounds. Turning and twisting round narrow hairpin bends, the procession sweeps into the broad street that leads past the handsome red Shire Hall and the great gloomy prison, until it finally reaches the wide, spacious racecourse by which the River Wear runs (Lawson, 1932, p. 126).

**Figure 1:**
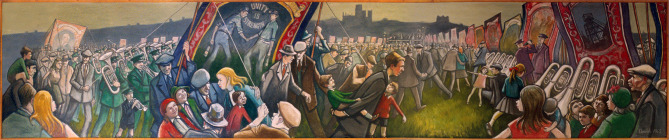
Norman Cornish, A Durham Miners' Gala Day Scene (1963).

A corollary of the resurgent interest in the Gala is the growing attention given to the miner artists of County Durham. The two greatest of these, Norman Cornish and Tom McGuinness (McManners and Wales, 1997, 2006; University Gallery Northumbria University, 2010) were concerned mainly with rendering the lives of the communities. Much of Cornish's work focus on his hometown of Spennymoor, while McGuinness' work paid special attention to the experience of underground work but ranged across subjects, themes, media. But both artists produced epic paintings of the Gala (McManners and Wales, 2002). Cornish was commissioned in 1962 to produce a mural for the newly opened Durham County Hall (see Figure 1). It depicts the scene on the Rcecourse, framed by the castle and cathedral. The mural itself measures 30′ 9^′′^ by 5′ 8^′′^, but Cornish also produced many accompanying sketches and studies. According to McManners and Wales, Cornish,

saw the banners as the sails of a galleon with three ‘waves' in the sea of people below them – the young people in the wave to the left represent and look toward the center, the future. The elderly folk to the right having deposited the past and they turn to the bold central banner bearing the slogan ‘Unity is Strength' – a symbol of the future (McManners and Wales, 2002, p. 120).

**Figure 2:**
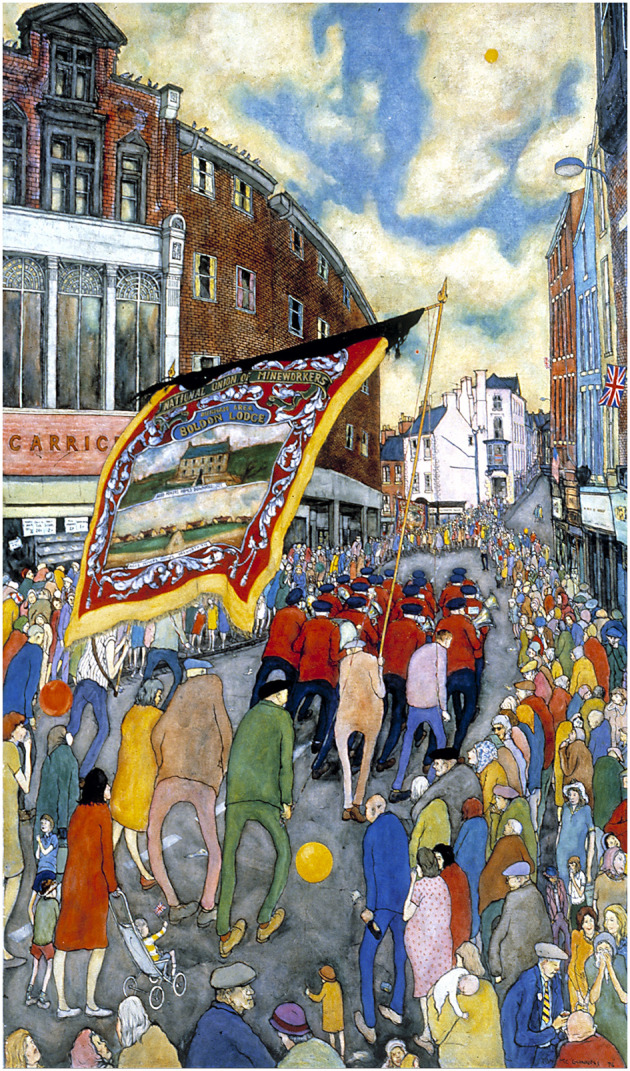
Tom McGuinness, Miners' Gala, 1976 (1977).

In 1968 McGuinness painted the Gala, representing the scene in front of the Royal County Hotel in Old Elvet, with the Labor dignitaries watching the passing bands and banners. He painted the Gala again in 1976 (Figure 2) capturing the scene as the Boldon banner and band leave the Market Place and wind through the narrows of Saddler Street toward Elvet Bridge and onto the Racecourse.

The poet Mary Wilson attended the Gala several times from the 1950s to the 1970s, accompanying her husband, the leader of the Labor Party and Prime Minster, Harold Wilson. Her poem, “The Durham Miners' Gala,” denotes the occasion in its post-war heyday. From her vantage point on the balcony of the County Hotel, where political dignitaries surveyed the parade, evokes the drama and its setting.

Suddenly we can hear the drums

Banging down the street;

And the brassy roaring of the bands,

And the rustle of marching feet,

And, with steadfast hands to hold them high

The silken banners go swaying by.

Bygone leaders and Bible scenes

Are woven on either side,

And the miners' lodge walks on before

Each banner, with solemn pride;

And the windows are full to see them go

And the cheers rise up from the crowd below.

…

Above the city's winding streets

The Cathedral stands alone,

And the sound of the bands is almost lost

In the lofty arches of stone.

The banners are blessed in the slanting sun –

And one more Gala Day is done.

(Wilson, 1970, p. 54–55).

Recalling the 1919 Gala in his unpublished memoir of 1971, *From Tyne to Tone* (Heslop, 1971), Harold Heslop (1898–1983), the Durham miner turned novelist, and a committed atheist and Communist (Fordham, 2009), suggests its location in Durham endows the event with a sacred quality:

In those days the Gala was a sight for all men to witness. The enormity of the proceedings outstripped the imagination. Perhaps it was the setting that lent privilege to the proletarian display. Maybe the vast mustering of the colliery tribes under the arches of the massive brick-built viaduct that spans the yonder part of the city and carries the great railway grants a piquancy to the subsequent proceedings. The booming of the drums provoking the attention of the tribes and then the double tap which unleashes the brazen sound into an almost dreamlike unreality and sets men and women marching. Repeated almost two hundred times, the resultant noise and slashings of color provoke an almost spiritual aura that hangs like a proud destiny over the immense beauty and rich dolour of the city.

The narrow streets – that were then – forced an intermingling of marchers and amused watchers. The crossing of the bridge over the Wear, that cowers like a coward within the ample shade of the great cliff that holds both castle and cathedral up to the arms of God, was always a strain lain upon the carriers of the banners. The passing over the bridge beneath the lovely scene evoked by tree-clad heights and glory-crowned buildings always evoked for me some strangely murmured benediction wailing softly into unreality. There is nothing so magnificent within Christendom that compares with the loveliness of Durham's cathedral. Ordinary vision, for they left it where it stands encompassing and encompassed by, its own earth, rising upwards to immortality like a prayer passing the lips of a woman suckling her babe.

It is in this cathedral which has softened the harsh lines of the men of coal every time they have ventured into the city to listen to the orators. It is never forgiving, never minatory. It watches them marching to their venue, and when all is over it beckons them back to their possession of their own lives. It is this half-church, half-refuge that softens the spirit after the pains of unremitting toil, and tempers the thundering of exhortation into croonings and beliefs (no page given)

Another version of the scene appears in his novel, *Goaf*, about the miners of “Darlstone” (a lightly fictionalized Durham),

“This day they had been to Darlstone. Wonderful old city! Glorious Vista! One day every year that gray old city is lit by the people. The miners. Their wives. Their children. To taste the deep drafts of endeavor one must journey to Darlstone on the day of the Gala. It is the day of the mineworkers. They come in their hundreds of thousands. They come as the people of separate villages, separate pits. They clutter proudly about their banners and follow their brass bands with a willingness born of great love. All the morning they march through the city, beneath the shadow of the cathedral that has gazed down from its heights to the silky-breasted river at its feet for centuries” (Heslop, 1935, p. 11).

The relationship of the city and the Gala is a recurring theme of the poet William Martin (Campbell, 2016). For Martin, the miners form a cohort of the *haliwerfolc*, the “people of the saint,” symbolically the followers of medieval St Cuthbert, whose remains are housed in the Cathedral. Cuthbert was a symbol of the region's identity and autonomy in the Middle Ages and responsible for numerous miracles. In “Durham Beatitude,” which memorializes the mining disaster at Easington in 1951, religious and mining language are interwoven,

What Kingdom

Without common feasting?

When they were seated

Silk banners on fellside

Friends after nettle-broth

Turned slogans into bread

By the poor for the poor

They taught themselves

…

Street children cry Cokerooso

Generations hop across

Spuggie-chorus crack

Cathedral-choir sing anthem

“Our feet shall stand

In thy gates O Jerusalem”

Larks rise with brass

Big Meeting last one for Eden.

(quoted in Durham Cathedral/Durham Miners' Association 2019).

The relationship between the mining communities and the ancient city, however, customarily was ambiguous. Leading families in the county who were coal owners traditionally had homes there. The Church of England was itself a major coal owner, although typically it leased its mineral rights. The relationship was signaled by the naming of the mine at Ferryhill, “Dean and Chapter”. Beynon and Austrin (1989) suggest that the decision of the DMA to hold the first Gala in the city was an act of resistance to the exclusion of working people from “Durham society.” Alongside the demands for material improvements was a “psychological dimension to Labor representation” and “an assertion of working-class self-respect” (Hayhurst, 2019, p. 3, 4). Thereafter, annually, the miners “occupied” the city each July. The slow accommodation of the Gala on the part of local elites was signified when in 1896 the DMA was invited by Bishop Westcott—“the Miners' Bishop” (Patrick, 2004)—to participate in a Cathedral service. The invitation established an annual tradition and became another part of the performance. According to his biographer, Peter Lee, the Primitive Methodist preacher who simultaneously presided over the Durham County Council and the DMA, fell under the spell of the Cathedral, “that magnificent thing of stone, oak and towers, built in old times when men would make a thing beautiful even if it took centuries to express their vision” (Lawson, 1949, p. 210).

In David Bean's novel, set in the early 1960s, the miners of “Sharbottle” gather at the Gala for the final time before the closure of their pit. The novel deals, among other things, with questions of industrial change, “modernization” and the fate of the Gala itself after the industry has gone. Ikey, the Lodge secretary and main character, has a love-hate relationship with the city at the center of the coalfield,

… because it had no pits of its own. A Cathedral and University city. A middle-class city looped off on three sides by a meander of its river, insulated from the world of miners by that same water and by the steep rocky banks which in its ancient founding time has insulated it from raiding armies. The place the monks bore St Cuthbert's corpse to for safety against the Danes. A more spectacular city than Heidelberg or Avignon, some had said. Well, Ikey had never been to either, had never set foot outside his own country, hardly outside his own county if it came to that, but he took the second-hand comparison for gospel and loved the tumbling, winding street and the stone bridges and the little sudden darting alleyways and flights of steps which would snatch you up from the main streets, from the traffic of the twentieth century, and wind you, fling you, up and up and up in spirals, back into the Middle Ages. But most of all he loved it on Gala Day, Big Meeting Day, because than the city was thrown open like a secret and the miners marched in and took over, made it their own (Bean, 1967, p. 59).

The recent rapid growth of Durham University has produced new local tensions. In November 2017, a University rugby club advertised a social event that required students to attend dressed as miners with “flat caps, filth and a general disregard for personal safety” and to “think pickaxes, think headlamps, think 12% unemployment in 1984.” Others were asked to dress as members of Thatcher's government, or as “working-class-beating-bobbies” to inject some “variety” to the event. The DMA objected. The event was canceled following media coverage and Durham University promised to enforce a code of conduct for students living in the local community that “aims to enhance students' sense of belonging” (Northern Echo, 2017). More broadly, the university's expansion plans generate tensions with the local communities that feel their needs are being overlooked (Northern Echo, 2019).

The Gala is deeply emplaced in Durham. It cannot be relocated in the way that investment, jobs and even people can be. To have meaning it can only take place within the confines of the medieval city. It has come to symbolize place and give expression to the identity of the communities than live in and around the city and to conflicts that are attached to these.

## Nostalgia, Memory, Heritage, Place

This essay has adopted an inductive approach in which the cultural record has been quarried in order to identify meanings of the Durham Gala. The central question has concerned why the Gala persists as a social and cultural phenomenon long after its apparent material basis has vanished. The preceding account has highlighted the complex, mutable and contested nature of the Gala. The Gala has been reinvented several times and has been dealing with the reality of industrial decline for half a century. The most recent reinvention—and resurgence—of the Gala signals another chapter in an ongoing story. Making sense of it requires us to think about the sociology of nostalgia, memory, heritage and place.

For some observers the Gala is an exercise in nostalgia for a world that should be left behind. It performs an outmoded ritual that should be laid to rest. But this is to misunderstand the nature and importance of nostalgia. Nostalgia affects longing for a home that no longer exists (Boym, 2011) and is a response to the pain and isolation produced by modernity. It is universal and permanent. Progressive critics conflate nostalgia with conservatism and display hostility to signs of yearning and loss and counterpose it to the values of cosmopolitanism. In the contemporary west, lines are drawn between the mobility and openness of progressive cities, with their “tropes of rootless reverie” (Bonnett, 2010, p. 172) and regressive “left behind” former industrial communities hampered by outmoded and insular attachments. Nostalgia, however, is not the antithesis of progress but its twin; it narrates modernity (Fritzsche, 2002). Sociology as a discipline has its origins in the search for lost organic communities. Modernity affects a more intensive and urgent relationship with loss (Davis, 1979). Nostalgia emerges from awareness of new eras, lost pasts, roads not taken. A yearning for better pasts recurs across times and cultures. Nineteenth century English radicals such as William Morris, Thomas Spence, and Robert Blatchford promoted, above all, a patriotic politics of conservation and resistance in the face of disruptive modernization that had far greater contemporary appeal than Marxism (Bonnett, 2010). Spence called for a return to the parish as the focus of a politics of mutual ownership (Knox, 1977). Twentieth century post-colonial struggles similarly focused on the rediscovery of traditions that had been swept aside by a western project of modernity (Nandy, 2011). Nostalgia is a social emotion, typically the product of current fears and anxieties, a way of maintaining solidarities in the face of disruption and pain and the erosion of hard-won social gains; economic dispossession “is the begetter of nostalgia, disenchantment the handmaiden of escapism” (Samuel, 2012, p. 261).

Dismissing the value of local attachments, (Amin, 2004) proposes, “a new politics of place” free of fixity and boundedness that recognizes the porosity of territories in the face of intensifying material and cultural flows.” Local cultural attachments are disparaged for promoting “a politics of local regard and local defense” and “a conservationist regional identity that can be profoundly closed and exclusionary” resting on “the scripting of a regional folk culture” (Amin, 2004, p. 35, 37). But the local struggles that are dismissed as regressive may also be in defense of hard-won, if partial and limited, social achievements (Tomaney, 2012). Across the Global North, as Seabrook (2005) has argued, the pain of passing of provincial life has been denied, “because everything that succeeded it has been tendentiously and insistently portrayed not as a mixture of the gains and losses that accompany all social change, but as irresistible progress toward a beckoning future over which dispute is not possible” (Seabrook, 2005, p. 237–238).

For the Left, according to Bonnett (2010), failure to acknowledge the power of nostalgia underlies its inability to connect with ordinary life and its concerns. Nostalgia gives expression to a sense of loss in communities that have experienced the demise of their traditional industries, loss of civic infrastructure and deteriorating social conditions. The decline of utopian visions in the twentieth century redirects attention to collective pasts that serve as repositories of inspiration for repressed identities and neglected claims because, the civic resources we need to master or contend with structural change, “are still to be found in the places and stories, memories and meaning, incidents and identities, that situate us in the world and give our lives their moral particularity” (Sandel, 1996, p. 349). The failure to grasp these insights underpins the crisis of left-wing political parties across the Global North and the rise of the populist Right.

Collective memory is crucial to the mobilization of civic resources (Olick et al., 2011). It is a social construction, shaped in part by the concerns of the present, and involves both continuity and change. Forgetting and erasure form components of the process. While it is individuals who remember they typically draw upon the social context to recollect and recreate the past. Hwalbachs (1992) asserts that it is in society that individuals recall, recognize, and localize their memories. Collective memories are emplaced and kept alive through activities that reproduce social bonds (Hebbert, 2005; Rowlands, 2007; Bennett, 2014, 2016). Periodic celebrations serve as focal points for the drama of civic remembrance. Rituals and representations establish connections between individuals and their cultural history and help form attachments to earlier periods (Davis, 1979). As autobiographical memory fades, historical memory involving reading, listening, and commemoration becomes more important. The past is stored and interpreted by social institutions with each generation counterposing its present to its own constructed past. Commemoration imaginatively enacts divergent historical paths. Anthropologists show how the past is employed by people to create a sense of identity (or identities) linked to myth and legend within established places in the landscape partially to legitimate the present, or “to mask change by stressing continuity” (Bender, 1993, p. 66). The way the past is memorialized is always changing. Durkheim (1995) understood that collective memory has a life of its own. Individuals cannot escape its consequences, hence the persistence of myth, ritual, tradition, heritage, although these rituals express exclusions and conflicts as well as organic solidarities.

Heritage conventionally is conceived in terms of physical objects, and academic debates have concerned the processes by which it is commodified. But underpinning the interest in heritage are basic human concerns. Hewison (1987, p. 45) notes that the urge to conserve the past reflects the impulse to preserve the self; “Without knowing where we have been it is difficult to know where we are going. Heritage is the foundation of individual and collective identity. Objects from the past are the source of significance as cultural symbols.” More recently, originating in the Global South, and reflecting the claims of indigenous peoples, attention has turned to the importance of intangible cultural heritage (UNESCO, 2003; Vecco, 2010; Lenzerini, 2011). “Authorized heritage discourses,” promoted by governments and their agencies, typically value artifacts, monuments and the nationally significant (in North East England, heritage artifacts for the era of coal is collected and displayed in museums at Beamish and Woodhorn; Vall, 2018). The worth of heritage is determined by cultural elites that value objects, while popular understandings are more likely to value memories, occasions, traditions. The promotion of intangible cultural heritage is often freighted with insurgent ambitions. Here the interest is in the safeguarding and management of a heritage that is mutable and part of “living culture.” But the task is to preserve cultures in ways that do not fossilize or trivialize it (Smith and Akagawa, 2009). Attachment to heritage, then, is not a form of “false consciousness” but a field of ideological, cultural and political conflict (Samuel, 2012). The notion of a living culture is captured in Williams (1978) elusive but fruitful notion of “structure of feeling,” which calls attention to how existing and new meanings of cultural formations coexist. The intangible aspects of a social formation have an evanescent character, traces of which linger long after the material basis for them has apparently disappeared.

Intangible cultural heritage is tied up with a sense of place. Place attachments—affective connections to a particular place—are a source of meaning for individuals and groups. Emotional connections invest meaning in places and shape behaviors. Place attachment reflects less longevity of residence and more the intensions to which a place gives rise. A sense of local belonging can be expressed individually or collectively but affects commitment to place. It can be attached to narratives of identity, but it may reflect practical commitments, investments, as well as longings and aspirations. Expressions of local belonging may embody a performative dimension which links individual and collective behavior and contributes to the formation of narratives of identity and the realization of attachments. Place is implicated in the formation of belonging in both its affective and political dimensions and is a focus for mutually constitutive relationships of attachment, loyalty, solidarity and sense of affinity which frame the processes by which a person becomes included in (or, excluded from) a socio-territorial collective and identified with it. Belonging affects matters of place through modes of boundary making, interwoven with symbolic and material spatialities (Tomaney, 2014). There are structural constraints on placemaking, so, “ordinary people seek to remake place and spatial relations to some extent, but not under conditions of their choosing” (Chari and Gidwani, 2005, p. 269). People work with the historical and cultural materials and the rituals that are at hand to enact a “performative discourse” that promotes a particular identity, although this can take diverse forms (Bourdieu, 1991, p. 233).

## Coda

The Gala speaks of a sense of loss on the part of communities in Durham in the aftermath of the end of coal mining. Communities can experience the loss of their traditional structures as a form of bereavement—“intense, deeply felt, and at times overwhelming” (Fried, 1966, p. 151). As Gordon (2008) attests, sociologists reckon with ghosts whose presence is painful, difficult and unsettling—the former coalfield haunts contemporary Durham. Losses go beyond immediate material dispossession to the realm of affective absences. The Gala exhibits many of the features of all popular fiestas, carnivals fêtes. Culture and heritage are retained in the process of staging them. The Gala gives expression to communal identities at the scale of the village and the county. The city of Durham provides the stage for the performance of this identity. The identities of Durham were always complex, contested, and subject to continuous transformation. Hard won solidarities and attendant social gains were challenged by the contraction of the coalfield but also by broader social changes, including shifting gender relations. Much of the nostalgia associated with the Gala concerns not so much the loss of industry and political power but a broader way of life and attachments that formed an affective infrastructure and imply cultural resistance in the face of processes of individualism, atomization, and privatization. While the Gala is typically understood through the lens of class, it speaks also of “vanishing grail of modern life: belonging” (Jones, 2019, p. 70). The search for community rather than a stage for revolutionary politics has always been the more important theme of the Gala.

What is the future of the Durham Miners' Gala? Its cultural afterlife is evident but the political context in which it occurs has changed radically. Once an arena for the Laborism of the Durham coalfield, this politics is in crisis. As a region which voted strongly in favor of Brexit, in the General Election of 2019, traditional Labor-voting seats in the heart of the former coalfield returned Conservative MPs for the first time. Explaining this outcome is beyond the scope of this paper. Structural and contingent factors are both important to any credible account, but it is clear that the old Labor tropes are losing their electoral appeal. The political infrastructure built up in villages over generations has gone, while social conditions have worsened for many. What is left of Laborism in County Durham draws on diminishing moral and civic capital accumulated during earlier generations. Yet, the contemporary political offer from a radicalized left-wing politics appears to have limited appeal in these communities. Today, the region abounds in unmet human needs and there is a yearning for community and belonging, and a space for a politics that recognizes this. The extraordinary contemporary revival of the Miners' Gala is powerful testament to this, even if its future is far from assured. The world that created the Gala was more divided internally, along religious, cultural and material lines, and more susceptible to competing narratives, than we realize today. Workplace solidarity and community cohesion were not bequeathed but were hard won, fragile, and partial. The achievements celebrated at past Galas were the product of compromise about political priorities that reconciled different interests and identities. The Gala, whatever its future, symbolizes almost 150 years of working-class endeavor in Durham.

## Data Availability Statement

All datasets analyzed for this study are cited in the article/supplementary materials.

## Author Contributions

The author confirms being the sole contributor of this work and has approved it for publication.

## Conflict of Interest

The author declares that the research was conducted in the absence of any commercial or financial relationships that could be construed as a potential conflict of interest.
